# Monocyte polarization in children with falciparum malaria: relationship to nitric oxide insufficiency and disease severity

**DOI:** 10.1038/srep29151

**Published:** 2016-07-07

**Authors:** J. Brice Weinberg, Alicia D. Volkheimer, Matthew P. Rubach, Salvatore M. Florence, Jackson P. Mukemba, Ayam R. Kalingonji, Charles Langelier, Youwei Chen, Margaret Bush, Tsin W. Yeo, Donald L. Granger, Nicholas M. Anstey, Esther D. Mwaikambo

**Affiliations:** 1 Duke University and V.A. Medical Centers, Durham, North Carolina, USA; 2 Hubert Kairuki Memorial University, Dar es Salaam, Tanzania; 3 University of California at San Francisco, CA, USA; 4 Menzies School of Health Research and Charles Darwin University, Darwin, Australia; 5 University of Utah and V.A. Medical Centers, Salt Lake City, Utah, USA

## Abstract

We earlier established that nitric oxide (NO) is protective against severe malaria and that arginine and NO levels are reduced in malaria patients. We now show that an M2-like blood monocyte phenotype is significantly associated with hypoargininemia, NO insufficiency, and disease severity in Tanzanian children with falciparum malaria. Compared to control children (n = 106), children with moderately severe (n = 77) and severe falciparum malaria (n = 129) had significantly higher mononuclear cell arginase 1 mRNA, protein, and enzyme activity; lower NOS2 mRNA; lower plasma arginine; and higher plasma IL-10, IL-13, and IL-4. In addition, monocyte CD206 and CD163 and plasma soluble CD163 were elevated. Multivariate logistic regression analysis revealed a significant correlation of risk of severe malaria with both plasma IL-10 and soluble CD163 levels. Monocyte M2 skewing likely contributes to NO bioinsufficiency in falciparum malaria in children. Treatments that reverse the M2 polarization may have potential as adjunctive treatment for malaria.

Falciparum malaria remains an important worldwide problem with over 200 million cases and an estimated 584,000 deaths each year^1^. We earlier established that nitric oxide (NO) is protective against severe malaria. Our detailed field studies in African children, and Indonesian children and adults have shown a close inverse association between malaria severity and mononuclear cell inducible NO synthase (NOS2) expression and systemic NO production^2^,^3^,^4^,^5^,^6^,^7^,^8^. We established that NO production in malaria is influenced by a variety of critical factors in the arginine-nitric oxide pathway, including reduced monocyte NOS2 expression, increased arginase activity and cell-free hemoglobin in plasma, and low levels of the NOS substrate arginine and the NOS cofactor tetrahydrobiopterin. While we have established that there are multiple mechanisms by which NO bioavailability is limited in malaria, we do not fully understand the underlying processes leading to NO bioinsufficiency and, in particular the impairment of NOS2 expression in severe malaria. The purpose of the current study was to determine the relationship of blood monocyte activation to hypoargininemia and low NO/NOS2 in Tanzanian children with malaria.

Investigators have noted that monocytes and macrophages can be activated *in vivo* or *in vitro* in response to infection with microbes or cytokines to display different phenotypes. Infection with Listeria monocytogenes or bacillus Calmette Guerin, or *in vitro* in treatment with cytokines such as IFN-γ and TNF causes “classical” (M1) activation with cells displaying enzymatic mechanisms of microbicidal activities including high NOS2-produced NO and NADPH oxidase-produced superoxide^9^,^10^,^11^,^12^. Arginase 1 is typically elevated in alternatively activated (M2) monocytes and macrophages *in vivo* or *in vitro* by treatment with factors such as IL-4, IL-13, IL-10, and TGF-ß^13^,^14^,^15^,^16^,^17^,^18^,^19^. M2 human mononuclear phagocytes are also characterized by expression of the mannose receptor (CD206) and the scavenger (hemoglobin-haptoglobin) receptor CD163^15^,^16^,^17^,^18^,^19^. Since M2 cells express very low NOS2/NO and produce high levels of arginase 1, an enzyme that depletes the NOS substrate arginine, we sought to characterize monocytes from children with falciparum malaria. We note that Tanzanian children with falciparum malaria have markedly increased PBMC arginase 1 and IL-10, reduced PBMC NOS2 and plasma arginine, and monocytes expressing markers of alternative activation (M2-like monocytes). The data indicates that this monocyte M2 skewing likely underlies NO bioinsufficiency in falciparum malaria in children.

## Results

### Clinical and laboratory data

We enrolled 312 subjects—106 HC, 77 MSM, 78 SM without cerebral malaria, and 51 with SM with CM (total of 129 SM) (Table 1). In the malaria patients, blood samples were drawn immediately on presentation (day 0). Healthy control individuals were significantly older and weighed more than those with malaria. Those with malaria had significant fever, tachypnea, tachycardia, and diastolic hypotension compared to HC children. Not all tests could be done in every patient because of necessarily low volumes of drawn blood in some individuals. Among patients with severe malaria, thirteen of 127 (10.2%) had severe anemia (Hb ≤ 5 gm/dL). Sixty seven of 129 (51.9%) had parasite counts >250,000/uL, 17/126 (13.5%) had pathologic deep breathing, and 48/126 (38.1%) had seizures. None of the patients died.

Malaria patients had significantly elevated monocyte and neutrophil counts, as well as decreased lymphocyte counts, and monocyte/lymphocyte ratios (Table 1). Platelet counts were decreased in all groups of malaria patients. Compared to HC, blood WBC and plasma creatinine were statistically increased in those with CM, but there was no difference in MSM patients or SM without CM. Plasma bicarbonate was significantly lower in SM and CM patients compared to HC, and plasma lactate was elevated in MSM, SM, and CM children. Parasite density was lower in MSM and CM compared to SM patients without CM (Table 1). Angiopoietin-2 levels were significantly higher in those with malaria; SM with CM patients had levels higher than those with MSM, and levels in CM patients were higher than those with SM without CM. This and other relevant data are displayed in detail in Table 1.

### Arginine and arginase

**Figure 1:**
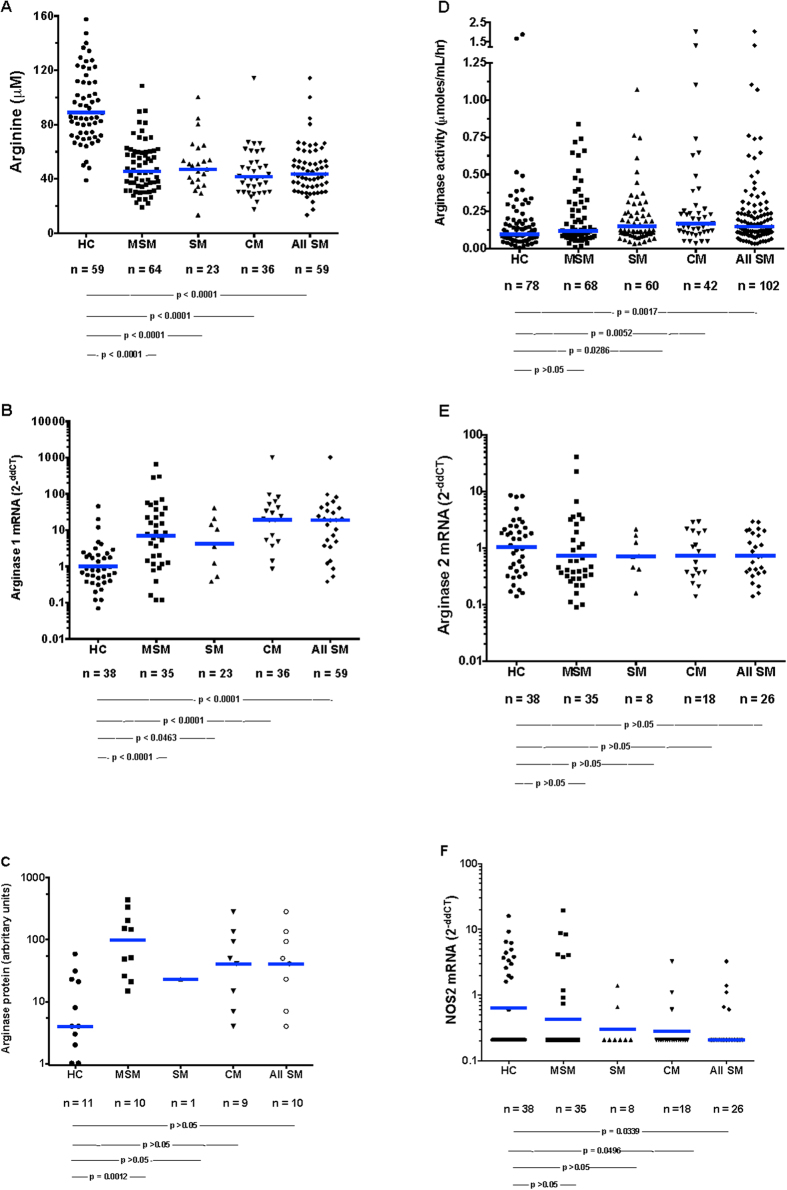
Plasma arginine (**A**), PBMC arginase 1 RNA (**B**) and protein (**C**), plasma arginase enzymatic activity (**D**), PBMC arginase 2 mRNA (**E**), and PBMC NOS2 RNA (**F**). The numbers of subjects and p values for the statistical pairwise comparisons (Mann-Whitney U) appear below the figures. The horizontal bars represent the geometric means.

As we have reported before, plasma arginine levels were significantly lower in all malaria groups compared to HC subjects at the time of presentation (day 0), but the levels were comparable in MSM, SM, and CM (Table 1 and Fig. 1A). Plasma arginase was significantly elevated in the SM and CM groups compared to HC (Fig. 1D). In Indonesian adults with falciparum malaria, we have noted elevated plasma arginase activity accompanied by increased plasma hemoglobin^20^. Since lysis of erythrocytes can release arginase 1 into plasma^21^, we considered that the intravascular hemolysis accompanying schizont rupture might cause high levels of plasma arginase with consequent degradation of arginine competing with NOS for the substrate arginine. However as opposed to Indonesian adults with malaria, plasma cell-free hemoglobin did not significantly differ across the spectrum of HC to CMs (Table 1). This makes it unlikely that a large proportion of arginase came from circulating lysed erythrocytes.

### Mononuclear phagocytes characteristics

Mononuclear phagocytes (monocytes and macrophages) can produce either arginase 1 or 2^22^. We considered that arginine was being diminished by arginase originating in mononuclear phagocytes. We did not have access to tissue macrophages, so we measured arginase 1 and arginase 2 mRNA in PBMC (Fig. 1). Arginase 1 (Fig. 1B) [but not arginase 2 (Fig. 1E)] was markedly (~30 fold) elevated in children with MSM, SM, and CM. Likewise, PBMC arginase 1 protein was higher in children with malaria than in HC children (Fig. 1C). We have noted low PBMC NOS2 protein and low NO production in children and adults with falciparum malaria^3^,^20^. In the current study, PBMC NOS2 mRNA was significantly (~4 fold) lower in children with SM compared to HC children (Fig. 1F).

**Figure 2:**
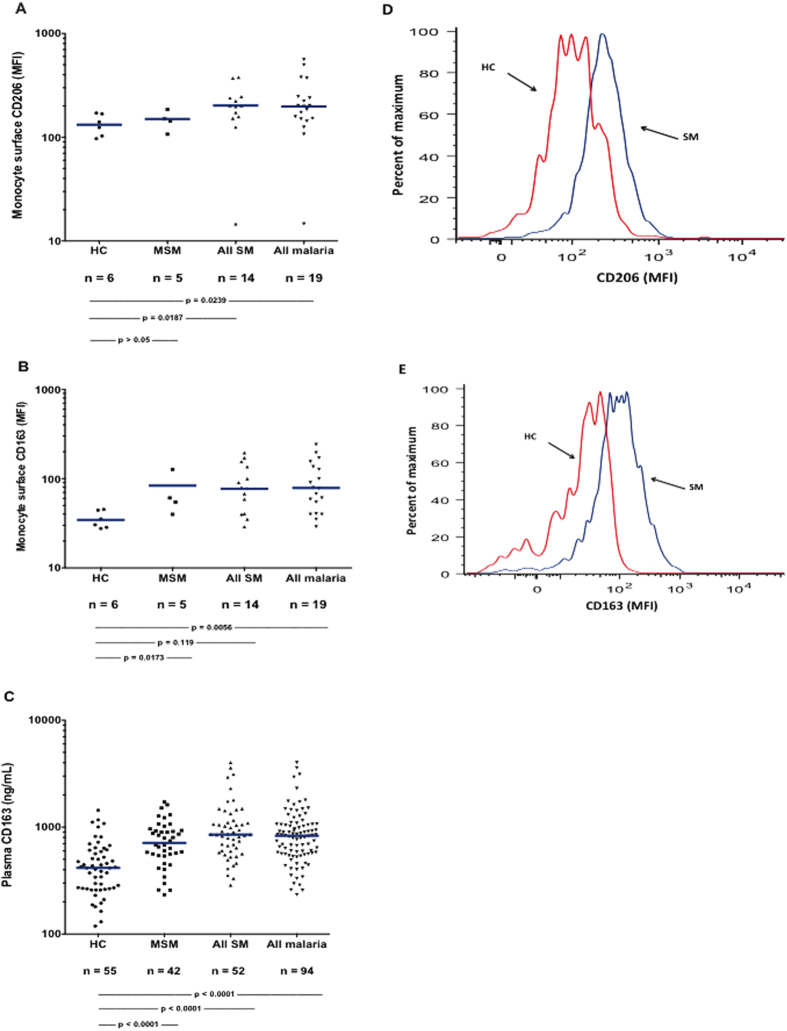
Blood monocyte cell surface CD163 and CD206, and plasma sCD163. Monocyte surface mean fluorescence intensities for CD206 (**A**) and CD163 (**B**); plasma soluble CD163 (**C**); and representative percent of maximal fluorescence for CD206 (**D**) and CD163 (**E**). The numbers of subjects and p values for the statistical pairwise comparisons (Mann-Whitney U) appear below the figures. The horizontal bars represent the geometric means.

Arginase 1 is typically elevated in alternatively activated (M2) monocytes and macrophages *in vivo*, or after *in vitro* treatment with factors such as IL-4, IL-13, IL-10, and TGF-ß^13^,^14^,^15^. M2 human mononuclear phagocytes are also characterized by expression of the mannose receptor (CD206), the scavenger (hemoglobin-haptoglobin) receptor CD163, and by elevated levels of arginase 1 and lower NOS2^15^,^16^,^17^,^18^,^19^. As measured by flow cytometry gating on monocytes, we found that blood monocytes from children with malaria had significantly higher levels of CD206 (Fig. 2A,D), and CD163 (Fig. 2B,E) than did those from HC children. As noted above, PBMC arginase 1 mRNA was markedly elevated and NOS2 lower in children with malaria (Fig. 1B,F). We also measured plasma soluble CD163 (sCD163), a soluble marker of mononuclear phagocyte M2 phenotype^23^: this was significantly elevated in children with MSM and SM (Fig. 2C).

### Plasma cytokines and chemokines

**Figure 3:**
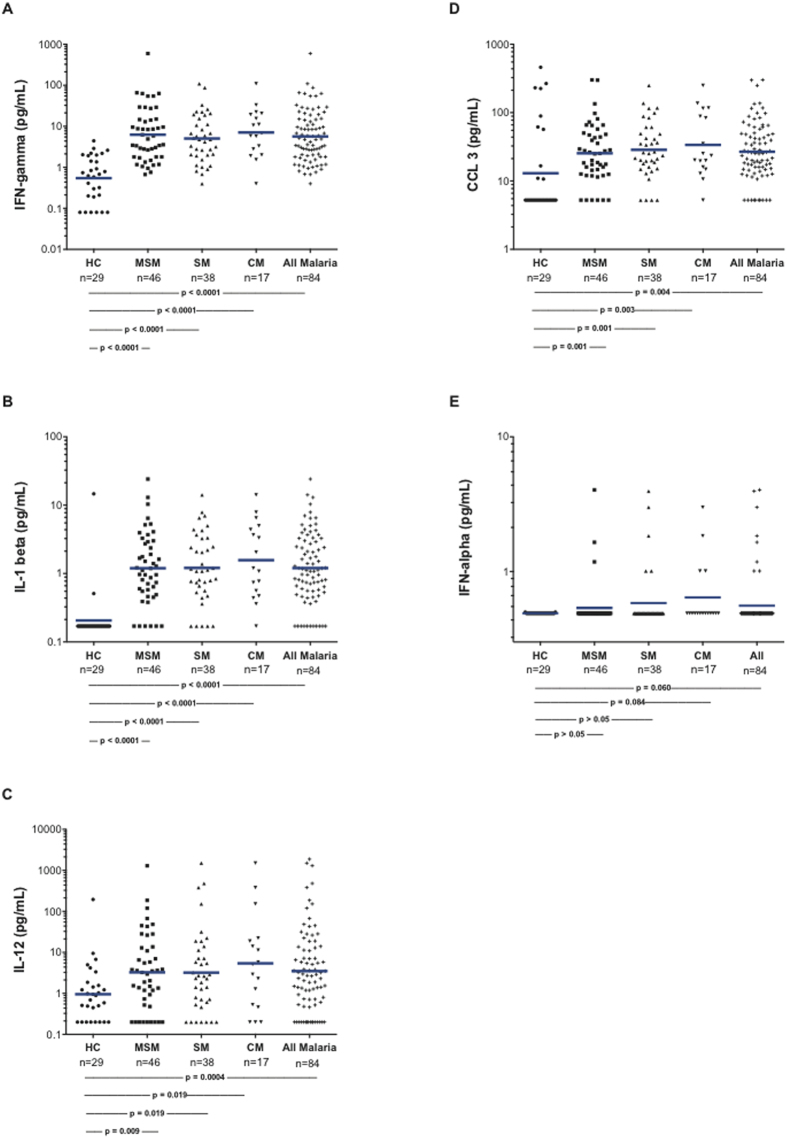
Plasma cytokines and chemokines generally considered typical of “M1” status. The numbers of subjects and p values for the statistical pairwise comparisons (Mann-Whitney U) appear below the figures. The horizontal bars represent the geometric means.

**Figure 4:**
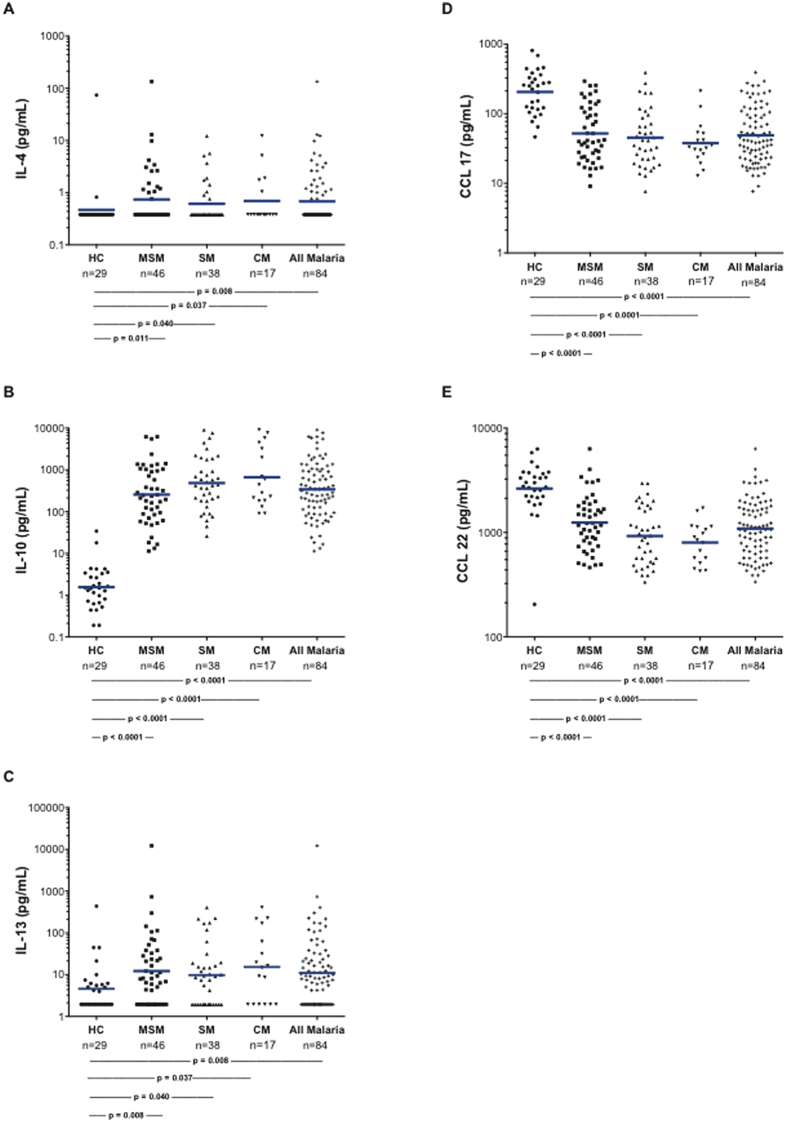
Plasma cytokines and chemokines generally considered typical of “M2” status. The numbers of subjects and p values for the statistical pairwise comparisons (Mann-Whitney U) appear below the figures. The horizontal bars represent the geometric means.

We measured plasma levels of selected cytokines and chemokines to assess potential soluble factors associated with M2 activation of the monocytes. Levels of the cytokines/chemokines characteristic of M1 monocytes (IFN-γ, IL-1 beta, IL-12, CCL3, and interferon-alpha) (Fig. 3) were elevated in the children with MSM and SM. Also, cytokines characteristic of M2 monocytes (IL-4, IL-10, and IL-13) were significantly elevated in MSM and SM (Fig. 4). IL-10 levels were markedly (300 to 5,000 fold) elevated in those with malaria. While levels of these cytokines and chemokines did not discriminate between the M1- and M2-like monocyte statuses, plasma IL-10 was significantly correlated with PBMC arginase 1 mRNA (r = 0.26, p = 0.0032). There were marked elevations of both M1 and M2 factors noted in SM and CM, and low levels of the M2-related chemokines in CCL17 and CCL22 in children with malaria.

### Correlations with markers of activation and disease severity

We tested relationships between plasma arginase activity, plasma sCD163, and plasma IL-10 with selected parameters of malaria disease activity and NO-related measures—HRP-2, angiopoietin-2, plasma lactate, plasma arginine, the arginine/ADMA ratio, blood hemoglobin, and blood platelets (Table 2). Among patients with malaria, arginase activity significantly correlated with angiopoietin-2, lactate, arginine, and the arginine/ADMA ratio. sCD163 correlated significantly with lactate and the arginine/ADMA ratio. And IL-10 correlated significantly with HRP-2, angiopoeitin-2, lactate, arginine, the arginine/ADMA ratio, and the platelet count in those with malaria (Table 2). In a backward stepwise multivariate logistic regression model for risk of severe malaria, we found that a unit increase in Log_10_ sCD163 was associated with a 4.68-fold (95% CI 1.31–16.68; p = 0.017) increase in odds of severe malaria (Supplementary Table 1). Similar models were constructed to examine significant associations between malaria disease severity and plasma IL-10 or arginase. A unit increase in Log_10_ IL-10 was associated with a 1.42-fold (95% CI 1.03–1.96; p = 0.032) increase in odds of severe malaria (Supplementary Table 2). Plasma arginase activity was not significantly associated with an increase in risk of disease severity (Supplementary Table 3).

## Discussion

Our data demonstrate that there is an M2-like activation monocyte phenotype in children with falciparum malaria, with high monocyte counts and plasma arginase levels, low arginine levels, and low monocyte NOS2, forming an important basis of the low NO bioavailability observed in malaria. We have shown that several different abnormalities apparently contribute to this state of low NO. We reported that adults and children with falciparum malaria have low NO levels and decreased PBMC NOS2 mRNA and protein expression^3^, low plasma arginine^4^, NOS2 promoter polymorphisms modulating NO production^5^, increased plasma arginase activity^3^, increased levels of the endogenous NOS inhibitor asymmetric dimethylarginine^6^,^24^, increased plasma levels of the NO-quencher hemoglobin^3^, and low levels of the NOS cofactor tetrahydrobiopterin^7^,^8^. This low NO status mediates endothelial dysfunction and likely contributes to the endothelial adhesion of parasitized RBC and impaired microvascular perfusion found in severe falciparum malaria.

**Figure 5:**
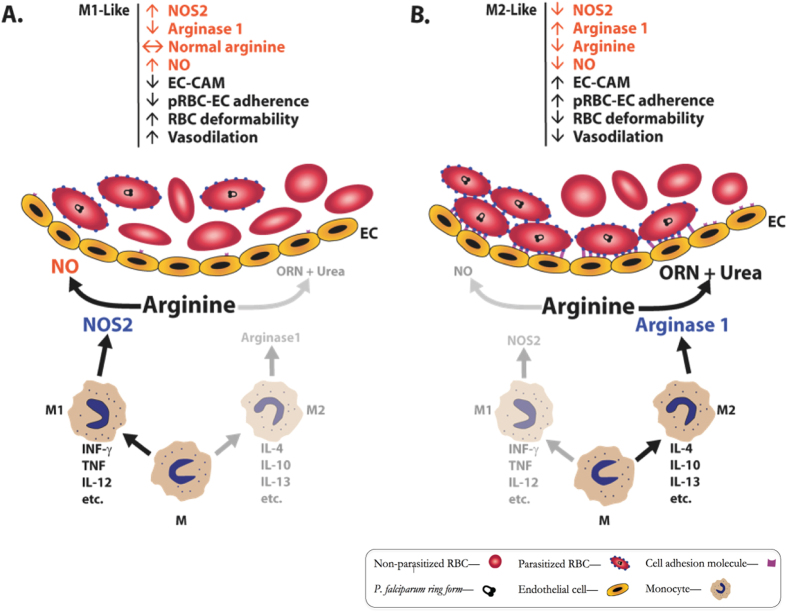
Schematic drawing depicting influence of M1 or M2 monocyte activation on arginase 1 and NOS2 expression, NO production, and adherence of parasitized RBC to endothelium. Monocytes (M) acted upon by various cytokines differentiate to (**A**) M1-like cells (“classically activated”) or (**B**) M2-like cells (“alternatively activated”). M1 cells can produce NOS2 that converts arginine to nitric oxide (NO) (**A**). M2 cells can produce arginase 1 that converts arginine to ornithine (ORN) and urea (**B**), causing depletion of arginine and reduction of NO. NO decreases expression of endothelial cell adhesion molecules (EC-CAM) (e.g., ICAM-1 and VCAM-1), and absence of NO or low NO causes increased CAM expression. RBC expressing *P. falciparum*-encoded proteins such as PfEMP1 bind to EC-CAM (**B**) causing blockage of blood flow and distal tissue ischemia. NO reduces EC-CAM expression, increases RBC deformability, and increases vascular reactivity.

Despite that accumulated knowledge, investigators have not been able to fully understand the basis of the low NOS2 levels and NO production in malaria. We show here that children with falciparum malaria have significantly increased levels of PBMC arginase 1 mRNA and protein, elevated plasma arginase activity, decreased PBMC NOS2 mRNA, and dramatically increased plasma levels of IL-10. Furthermore, blood monocytes from malaria patients have increased levels of the monocyte scavenger receptor CD163 and the mannose receptor CD206, and high plasma levels of soluble CD163. These findings and this monocyte phenotype are comparable to those noted in alternatively activated (M2) monocytes. Based on these findings, we conclude that these plasma cytokine/chemokine and PBMC/monocyte alterations characterize a unique malaria monocyte activation phenotype that likely contributes to the NO bioinsufficiency that is significantly associated with the development of severe malaria. Figure 5 schematically depicts the influence of M1 and M2 monocyte activation on NO bioavailability and adherence of parasitized RBC to endothelium, a critical pathophysiological mechanism of severe disease in malaria. This M2-like malaria activation phenotype may thus contribute to vascular dysfunction leading to worsening illness. As noted below, other researchers have emphasized that in human clinical medicine, it is unusual to encounter disorders in which the cells are all M1 or all M2; rather the phenotypes are more likely “mixed” or “in transition”^14^,^25^.

Macrophage activation was initially characterized in mouse peritoneal macrophages based on “activation” *in vivo* during infection with intracellular organisms such as *Listeria monocytogenes* or bacillus Calmette Guerin^9^,^26^. The activated cells mediated resistance to subsequent infection with unrelated organisms and to tumor cell growth. Subsequent studies documented that the activation was mediated by cytokines (e.g., IFN-γ and TNF) and endotoxin^10^,^11^,^12^. This is now generally termed the “classical” (M1) pathway of activation. M1 mononuclear phagocytes acquire enzymatic mechanisms of microbicidal activities including NOS-produced NO and NADPH oxidase-generated superoxide^10^,^11^,^12^,^27^.

Later a second pathway of “alternative” (M2) activated mononuclear phagocytes was characterized^15^,^16^,^17^,^18^,^19^. This M2 response corresponds roughly to Th2-acquired immunity driven by IL-4 and IL-13. Some have divided the M2 category based on the specific activating stimuli^14^, and *in vitro* studies with human monocytes have established numerous conditions for activating these cells to several different phenotypes^28^. M2 cells decrease the classical activation response preventing tissue injury, and they participate in wound healing and granuloma formation with multinucleated mononuclear phagocyte fusion. M2 mononuclear phagocytes in humans are characterized by high expression of the mannose receptor CD206, the hemoglobin-haptoglobin scavenger receptor CD163, high arginase 1, and low NOS2. M1 and M2 mononuclear phagocytes secrete a spectrum of cytokines and chemokines that are responsible for the cell types characteristic of their respective inflammatory infiltrates. M2 mononuclear phagocyte transition states can be along a continuum^15^,^16^,^17^,^18^,^19^,^28^. There are distinct differences in mouse and human mononuclear phagocytes relative to M1 and M2 activation paths and cellular characteristics^11^,^14^,^29^. Researchers have noted that in human clinical medicine, it is unusual to encounter disorders in which the cells are all M1 or all M2; rather the phenotypes are more likely “mixed” or “in transition”^14^,^25^.

RBC contain arginase 1, and RBC lysis releases arginase 1 into plasma. We have noted high levels of erythrocyte-free hemoglobin in plasma of Indonesian adults with falciparum malaria^20^. However, our current work shows that plasma cell-free hemoglobin is only modestly elevated in children with CM and not significantly elevated/altered in the other groups. This makes it unlikely that a large proportion of plasma arginase comes from lysed erythrocytes. We suspect that the elevated plasma arginase activity in children with severe malaria primarily arises primarily from activated mononuclear phagocytes (monocytes and tissue macrophages) expressing arginase 1. However, plasmodia can also produce a functional arginase^30^ that could contribute to the increased levels of plasma arginase from host monocytes that we observe.

The increased polarization towards the M2 phenotype and the increased arginase activity may also affect the immune response in malaria by altering the metabolism substrates available to lymphocytes^31^ and other immune cells, as well as by hypoargininemia which can increase regulatory T cell expression^32^. M2 macrophages rely on oxidative metabolism, while M1 cells obtain energy via glycolysis^33^. This may be a possible explanation for the otherwise unexplained increase in oxygen consumption that we have noted in both children and adults with malaria^34^,^35^.

CD163 is a receptor for hemoglobin-haptoglobin complexes. Mononuclear phagocyte expression of CD163 can be stimulated by free hemoglobin, heme, hemozoin, IL-10, IL-4, and glucocortioids^23^,^36^. Others have demonstrated elevation of sCD163 in falciparum^37^,^38^ and vivax malaria patients^39^. Levels of CD163 correlate positively with severity of diseases^39^ and inversely with hemoglobin levels^38^. We further demonstrate a significant correlation between this M2 marker sCD163 and other parameters that are known to correlate with falciparum disease severity (Table 2). Multivariate regression analyses demonstrate that elevated levels of plasma sCD163 as well as IL-10 are independent parameters that significantly increase the odds of severe malaria by 4.7 fold and 1.2 fold, respectively (Supplementary Tables 1 and 2). sCD163 is a specific marker for anti-inflammatory mononuclear phagocytes^23^. Levels are known to be elevated in patients with acute bacterial infections, and are significantly related to prognosis and death in these patients^40^. Likewise, sCD163 plasma levels are elevated in patients with tuberculosis [and are predictive for survival^41^], as well as in patients with hematopoietic malignancies^42^, atherosclerosis-coronary artery disease^43^, and inflammatory diseases such as rheumatoid arthritis^44^.

IL-10 and lactic acid levels are of particular interest. Both IL-10^45^ and lactic acid^46^ can decrease classical (M1) mononuclear phagocyte activation and reduce NOS2 expression, and lead to M2 monocyte activation. We note that blood lactate correlates with plasma arginase activity, IL-10, and sCD163 in malaria, consistent with a role of lactate in M2 activation. Colegio and others showed that tumor-derived lactic acid induces expression of macrophage VEGF and arginase, and M2-like polarization of macrophages through mechanisms involving hypoxia inducing factor 1 (HIF-1) alpha^46^. They also found that arginase 1 produced by the M2-like macrophages influences tumor growth. IL-10 in general suppresses macrophage production of cytokines such as TNF and IL-1^47^,^48^,^49^,^50^. In addition, IL-10 inhibits transcription of NOS2 mRNA and NO production^50^. We previously showed that plasma IL-10 is markedly elevated in children with falciparum malaria^3^. We demonstrate here that plasma IL-10 correlates significantly with both M2 phenotype and disease severity, with IL-10 levels markedly elevated in MSM and CM (CM > MSM).

In studies of a malaria co-infection model in mice, Lokken and co-workers noted that animals infected with *Plasmodium yoellii* have reduced ability to resist infection with non-typhoid *Salmonella typhimurium*. This is associated with high levels of IL-10 and presence of macrophages with certain characteristics of alternative activation^51^. However, similar to our findings, the macrophages had a mixed phenotype, with elevated TNF and IFN-gamma (M1 markers), as well as elevated IL-10 and other markers of M2 mouse macrophages (e.g., Fizz1, Ym1, arginase 1, and CD206). They clearly demonstrated that IL-10 produced by macrophages (and acting on macrophages) was the mediator of the reduced resistance to non-*S. typhimurium* infection in the mice. In other work, Antonelli and others using a different classification for monocytes showed that patients with *P. vivax* infection have “activated monocytes” in the blood defined as CD14+CD16+ (classical), CD14+CD16+ (inflammatory), and CD14loCD16+ (patrolling) cells^52^. They found that classical and inflammatory monocytes were the primary source of pro-inflammatory cytokines. CD14+CD16+ monocytes were more efficient in phagocytizing *P. vivax*-infected reticulocytes, which induced them to produce high levels of TNF-α and reactive oxygen species. Teirlinck and co-workers studied antigen presenting blood cells in normal volunteers intradermally infected with *P. falciparum* sporozoites^53^. They noted increased numbers of subsets of monocytes and CD16 positive myeloid dendritic cells during the blood stage of infection.

Martinez and Gordon^14^ and Murray *et al.*^25^ have emphasized that it is unusual to find “pure” M1 or M2 mononuclear phagocyte activation in human disease. M1 and M2 signatures do not necessarily exclude each other, and they often coexist, depending in part on the balance of activating and inhibiting stimuli in the local environment. Indeed, we see monocytes with a mixed phenotype, displaying some M2 and some M1 characteristics in our study participants with malaria. However, PBMC arginase 1, and plasma IL-10 and sCD163 levels are elevated to extreme degrees. Notably of the chemokines and cytokines we measured, CCL17 [“thymus and activation related chemokine” (TARC)] and CCL22 [“macrophage derived chemokine” (MDC)] are the only two that are low in malaria. In agreement with our data, Wangala and others reported earlier that CCL22 is lower in SM than in HC individuals^54^. The precise meaning of the lower levels of CCL17 and CCL22 in malaria is currently unknown, but it does underscore the complexity of simply analyzing plasma chemokines as markers of mononuclear phagocyte activation status.

In this report, we have studied healthy children, children with moderately severe malaria, and children with severe malaria. Future studies of children with asymptomatic parasitemia and children with malaria not requiring hospitalization (“uncomplicated malaria”) will likely provide additional important information relevant to work that we report here. Also, it is possible that helminth infestation may have affected our findings. We did not test for helminth infestation in any of the children. The influence of helminth infestation on malaria severity is controversial—depending on the helminth and the publication, some have reported that helminthiasis may either reduce or enhance malaria severity^55^,^56^. In addition, helminthiasis may be associated with M2 activation of macrophages^56^,^57^. Future studies dissecting the influence of helminth infestation on monocyte phenotype and malaria severity will be helpful.

In summary, the M2-like activation of monocytes in malaria leads to hypoargininemia and NO insufficiency. It is important to note that there are a variety of agents that can prevent activation to M2, or reverse M2 activation to unactivated cells or M1 cells. These include IFN-γ^10^,^15^, IL-12^15^, LPS^10^,^15^, GM-CSF^58^, bisphosphonates^59^, and statins^60^. Results from our prior work demonstrated that administration of ultra-low doses of falciparum infected erythrocytes to normal volunteers induced immunity to malaria, and that this immunity correlated with a T cell proliferative response with high levels of IFN-γ, low levels of IL-10, and high levels of blood mononuclear cell NOS activity^61^. This suggests that an IFN-γ in humans with malaria induces M1 activation of monocytes and that this is associated with increased resistance to malaria.

In addition to reducing malaria severity, studies in mice have shown that it is possible that some of these agents (e.g., IFN-γ) could, through pro-inflammatory effects, make the malaria worse^62^. Careful experimental testing of certain agents that modulate monocyte activation should be considered for eventual development as adjunctive treatments for malaria in hopes of preventing/reversing the M2 monocyte/macrophage activation status, increasing NOS2 expression, decreasing IL-10, enhancing NO bioavailability, and reducing malaria severity.

## Methods

We obtained written informed consent for participation from parents or guardians of all subjects. In Dar es Salaam, Tanzania, children between ages 4 and 8 years were enrolled from Amana or Mwananyamala District Hospitals, or the Hubert Kairuki Memorial University Memorial University (HKMU). The study was approved by the Ethics Committees/IRBs of HKMU, the Tanzanian National Institute for Medical Research, Duke University Medical Center, University of Utah, Menzies School of Health Research, and the Salt Lake City and Durham V.A. Medical Centers. Methods were carried out in accordance with the approved guidelines. Subjects were enrolled from 2008 through 2014.

Healthy controls (HC) were defined as unrelated children who were subjectively well with no history of fever in the preceding 48 hours, no parasitemia by microscopy, and no concurrent illness or medication. Moderately severe malaria (MSM) children were defined as those with fever or a history of fever in the past 48 hours; with >2,500 asexual falciparum parasites/μL (a parasitemia threshold above that predicting clinical disease elsewhere in coastal East Africa); no other etiology identified, and requiring inpatient parenteral therapy because of inability to tolerate oral therapy, but exhibiting no WHO warning signs or criteria for severe malaria^20^,^63^. Severe malaria (SM) children were defined as those having falciparum parasitemia (>2,500 parasites/μL) and ≥1 modified WHO criteria of severity^63^, cerebral malaria (CM; Blantyre coma score <3); or respiratory distress/metabolic acidosis defined as deep breathing and/or laboratory-confirmed acidosis (venous bicarbonate <15 mmol/L), with no other evident cause; and (in those with coma) CSF white cells <50/μL^64^. Patients were treated with anti-malarials including quinine or artemisinins and antibiotics using standard national protocols prevailing at the time of the study. In total, 97% of the MSM and SM patients were treated with parenteral quinine on admission, and 3% were given artemether.

Heparinized blood was collected and processed rapidly, and counted on an automated blood counting machine (Beckman-Coulter, Model Act 10). Hemoglobin, biochemistry, acid base parameters, and lactate were measured with a bedside analyzer (i-STAT Corp). WBC differentials were determined manually by visual examination of stained blood films. Plasma levels of arginine were quantitated by HPLC as previously described^20^; histidine-rich protein-2, and angiopoietin-2 were quantified by ELISA^24^; and plasma arginase activity was measured using a radiometric assay we have described before^20^. Cell-free plasma hemoglobin^65^ and sCD163 were measured by ELISA (R&D Systems, Minneapolis, MN). We determined plasma cytokine and chemokine levels using ELISA with materials from Meso Scale Discovery (Rockville, MD). The lower limits of detection were as follows: IFN-α (0.761 pg/mL), IFN-γ (0.097 pg/mL), IL-1β (0.265 pg/mL), IL-4 (0.292 pg/mL), IL-10 (0.308 pg/mL), IL-12 p70 (0.369 pg/mL), IL-13 (2.99 pg/mL), CCL22 (162 pg/mL), CCL4 (7.93 pg/mL), CCL17 (4.75 pg/mL).

We isolated PBMC using standard methods with density gradient sedimentation with ficoll/Hypaque. Cellular RNA and protein were extracted using the “All Prep” kit (Qiagen, Germantown, MD). We subjected RNA to real-time reverse transcriptase-polymerase chain reaction (RT-PCR) analysis as we have described before^7^. We used the equivalent of 100 ng RNA per reaction. Stratagene VILO kit was used for the RT reaction, and the Roche Faststart universal probe master kit for PCR (Roche Applied Science, San Francisco, CA). Primer/probe sets arginase 1, arginase 2, NOS2a, GAPDH, and HPRT1 were designed by the Roche universal probe library application (Supplementary Table 4)^7^. Primers were then made by IDT. Probes were from the Roche universal probe library (Roche Applied Science, San Francisco, CA) (http://universalprobelibrary.com). The quantitative PCR reactions were done using an ABI7300 machine. Samples were run in triplicate, or in duplicate if sample amount was limiting. We selected GAPDH and HPRT1 genes as endogenous control genes. The average crossing threshold (Ct) of the endogenous controls for each sample was used in the dCt calculation. We used each sample dCt and the averaged healthy control dCt or individual ddCt values. For group ddCt values, we used the average dCt for each group to calculate ddCt for the group. The data are expressed as 2-ddCt, which is the fold change as compared to the average of the healthy controls. Surface antigens on blood cells were detected with a Becton-Dickinson LSR-II cytometer using the following antibodies: CD14 (Becton-Dickinson, San Jose, CA), CD163 (BioLegend, San Diego, CA), and CD206 (BioLegend, San Diego, CA). We determined cell viability with Aqua Vital Dye (Life Tech, Grand Island, NY), and we gated Vital Dye negative and CD14 positive cells as monocytes. We quantified PBMC arginase 1 protein by immunoblot with densitometry using an affinity purified polyclonal, goat anti-human arginase 1 antibody (Santa Cruz Biotechnology, Santa Cruz, CA).

Statistical analyses were performed using STATA software (version 13.0; StataCorp, College Station, TX). Results are presented as mean with standard deviation for normally distributed continuous variables or median with interquartile range for variables with non-parametric distribution. For continuous variables with normal distributions, differences across groups were compared by ANOVA with Bonferroni adjustment for multiple comparisons, and differences between groups were compared using Student’s t-test. For continuous variables with a nonparametric distribution, we compared differences across groups with the Kruskal-Wallis test and differences between groups with the Wilcoxon rank-sum test. Correlation between variables with non-parametric distributions was assessed using Spearman correlation coefficients. Differences in proportions between groups were assessed with Chi-square test. We did multivariate linear regression analyses to control for age, weight and sex when comparing concentrations of chemokines and cytokines among the various clinical groups.

Among patients with malaria, backward stepwise multivariate logistic regression was performed to determine whether plasma soluble CD163, plasma IL-10, or plasma arginase activity were independently associated with disease severity. Bivariate logistic regression was used for analysis of soluble CD163, IL-10, and arginase activity along with other cytokines/chemokines. Additional variables included in the multivariate model based on biological plausibility included hemoglobin and absolute monocyte count (based on their established biological relationships to analytes in question); lactate (a variable known to be associated with malaria severity); age, weight and sex (since these may interact with sCD163, IL-10, or arginase activity measurements in children). Other established biomarkers of disease severity, such as HRP-2 or angiopoietin-2, were not included in the final multivariate logistic regression model due to the limited number of observations (i.e., limited number of malaria patients with measurements for all variables). Similarly, a combined summary multivariate logistic regression including sCD163, IL-10, arginase activity, and other biologically plausible variables was not feasible because of the limited number of observations that included all variables. Continuous variables with non-parametric distributions were log-transformed to meet normality assumptions for use in the logistic regression models. We assessed goodness-of-fit with the Hosmer-Lemeshow test. A two-sided p-value < 0.05 was employed as the cut-off for statistical significance throughout.

## Additional Information

**How to cite this article**: Weinberg, J. B. *et al.* Monocyte polarization in children with falciparum malaria: relationship to nitric oxide insufficiency and disease severity. *Sci. Rep.* **6**, 29151; doi: 10.1038/srep29151 (2016).

## Supplementary Material

Supplementary Information

## Figures and Tables

**Table 1:** Patient characteristics and laboratory data according to clinical status.

	Healthy control (HC)	Moderately severe malaria (MSM)	Severe malaria without cerebral malaria (SM)	Cerebral malaria (CM)	p values
Total number	106	77	78	51	
Age (years)^a^	7.4 (1.8) (n = 104)	5.2 (1.7) (n = 76)	5.4 (1.7) (n = 77)	4.8 (1.3) (n = 51)	ANOVA— <0.0001 HC vs MSM— <0.0001 HC vs SM— <0.0001 HC vs CM— <0.0001MSM vs SM— NS MSM vs CM— NS SM vs CM— 0.0272
Males (percent of total)^b^	48 (46)	45 (59)	38 (48)	32 (63)	P = 0.110
Weight (Kg)^a^	23.0 (6.2) (n = 106)	17.7 (4.0) (n = 76)	17.3 (3.6) (n = 76)	17.0 (4.6) (n = 51)	ANOVA— <0.0001 HC vs MSM— <0.0001 HC vs SM— <0.0001 HC vs CM— <0.0001 MSM vs SM— NS MSM vs CM— NS SM vs CM— NS
Temperature (°C)	36.6 (36.5–36.8) (n = 106)	37.4 (36.7–38.5) (n = 76)	37.4 (36.7–38.0) (n = 77)	38.0 (36.9–38.8) (n = 51)	Kruskal Wallis— <0.0001 HC vs MSM— <0.0001 HC vs SM— <0.0001 HC vs CM— <0.0001 MSM vs SM— NS MSM vs CM—NS SM vs CM— 0.0415
Heart rate (beats/min)	91 (86–97) (n = 106)	110 (100–120) (n = 76)	110 (100–120) (n = 77)	120 (105–140) (n = 51)	Kruskal Wallis— <0.0001 HC vs MSM— <0.0001 HC vs SM— <0.0001 HC vs CM— <0.0001 MSM vs SM— NS MSM vs CM— 0.0033 SM vs CM— 0.0201
Respiratory rate (breaths/min)	25 (23.0–26.0) (n = 101)	29.5 (26.0–34.0) (n = 74)	31.0 (28.0–38.0) (n = 77)	38.0 (30.0–48.0) (n = 51)	Kruskal Wallis— <0.0001 HC vs MSM— <0.0001 HC vs SM— <0.0001 HC vs CM— <0.0001 MSM vs SM— 0.0188 MSM vs CM— <0.0001 SM vs CM— 0.0144
Systolic blood pressure (mm Hg)^a^	92 (6.6) (n = 104)	93 (6.9) (n = 70)	91 (5.7) (n = 75)	92 (8.2) (n = 49)	ANOVA—NS HC vs MSM— NS HC vs SM—NS HC vs CM— NS MSM vs SM— 0.0498 MSM vs CM— NS SM vs CM— NS
Diastolic blood pressure (mm Hg)^a^	60 (2.9) (n = 104)	58 (4.8) (n = 70)	56.4 (5.3) (n = 75)	56 (11.1) (n = 49)	ANOVA—0.0001 HC vs MSM— 0.0004 HC vs SM— <0.0001 HC vs CM— 0.0002 MSM vs SM— 0.0438 MSM vs CM— NS SM vs CM— NS
Hemoglobin (gm/dL)^a^	12.1 (1.3) (n = 104)	8.8 (1.9) (n = 75)	8.4 (2.2) (n = 77)	7.3 (2.3) (n = 50)	ANOVA— <0.0001 HC vs MSM— <0.0001 HC vs SM— <0.0001 HC vs CM— <0.0001 MSM vs SM— NS MSM vs CM— 0.0001 SM vs CM—0.0031
WBC (x10^9^/L)	6.7 (5.7–8.2) (n = 105)	7.2 (6.0–9.6) (n = 74)	7.9 (5.9–11.1) (n = 77)	8.3 (6.9–9.9) (n = 51)	Kruskal Wallis0.0035 HC vs MSM— NS HC vs SM— 0.0147 HC vs CM— 0.0006 MSM vs SM— NS MSM vs CM— NS SM vs CM— NS
Platelet (x10^9^/L)	338 (226–409) (n = 106)	113 (71–163) (n = 74)	80 (53–126) (n = 77)	73 (54–104) (n = 48)	Kruskal Wallis— 0.0001 HC vs MSM— <0.0001 HC vs SM— <0.0001 HC vs CM— <0.0001 MSM vs SM— 0.0229 MSM vs CM— 0.0013 SM vs CM— NS
Absolute monocyte count (x10^9^/L)	0.51 (0.38–0.61) (n = 78)	0.60 (0.44–0.82) (n = 66)	0.67 (0.48–1.06) (n = 73)	0.69 (0.49–0.99) (n = 33)	Kruskal Wallis—0.0001 HC vs MSM— 0.0062 HC vs SM— 0.0001 HC vs CM— 0.0003 MSM vs SM— NS MSM vs CM— NS SM vs CM— NS
Absolute neutrophil count (x10^9^/L)	2.6 (1.9–3.5) (n = 76)	3.7 (2.9–5.5) (n = 65)	4.4 (3.2–5.8) (n = 73)	4.8 (2.9–5.9) (n = 48)	Kruskal Wallis— <0.0001 HC vs MSM— <0.0001 HC vs SM— <0.0001 HC vs CM— <0.0001 MSM vs SM— NS MSM vs CM— NS SM vs CM— NS
Absolute lymphocyte count (x10^9^/L)	2.9 (2.3–3.2) (n = 78)	2.4 (1.7–3.1) (n = 66)	2.1 (1.5–2.9) (n = 73)	2.2 (1.7–3.0) (n = 33)	Kruskal Wallis 0.0074 HC vs MSM— 0.0081 HC vs SM— 0.0010 HC vs CM— 0.0012 MSM vs SM— NS MSM vs CM— NS SM vs CM— NS
Monocyte to lymphocyte ratio	0.18 (0.14–0.24) (n = 77)	0.28 (0.20–0.37) (n = 66)	0.32 (0.23–0.41) (n = 73)	0.31 (0.24–0.43) (n = 33)	Kruskal Wallis— <0.0001 HC vs MSM— <0.0001 HC vs SM— <0.0001 HC vs CM— <0.0001 MSM vs SM— 0.0210 MSM vs CM—NS SM vs CM— NS
Creatinine (mg/dL)	0.4 (0.4–0.5) (n = 53)	0.5 (0.4–0.6) (n = 44)	0.4 (0.3–0.5) (n = 46)	0.6 (0.4–0.08) (n = 32)	Kruskal Wallis— <0.0001 HC vs MSM— NS HC vs SM— 0.0386 HC vs CM— 0.0006 MSM vs SM—NS MSM vs CM— 0.0030 SM vs CM— 0.0001
Blood lactate (mmol/L)	1.9 (1.4–2.5) (n = 76)	2.7 (1.8–3.6) (n = 55)	2.8 (2.0–4.1) (n = 59)	2.9 (2.0–4.7) (n = 41)	Kruskal Wallis— <0.0001 HC vs MSM— 0.0001 HC vs SM— <0.0001 HC vs CM— <0.0001 MSM vs SM— NS MSM vs CM— NS SM vs CM— NS
Bicarbonate (mmol/L)^a^	22.5 (2.9) (n = 50)	22.3 (3.5) (n = 58)	21.1 (3.1) (n = 59)	19.9 (4.4)) (n = 43)	ANOVA—0.0005 HC vs MSM— NS HC vs SM— 0.0049 HC vs CM— 0.0003 MSM vs SM—0.0203 MSM vs CM— 0.0013 SM vs CM— NS
Parasite density (per uL)	0 (0-0) (n = 106)	90,280 (49,060–144,000) (n = 76)	286,920 (203,820–400,000) (n = 78)	177,440 (49,480–312,000) (n = 49)	Kruskal Wallis— <0.0001 HC vs MSM— N/A HC vs SM— N/A HC vs CM— N/A MSM vs SM— <0.0001 MSM vs CM— 0.0149 SM vs CM— 0.0004
Histidine rich protein-2 (ng/mL)	0.3 (0.3–0.3) (n = 60)	163.4 (26.6–917.6) (n = 65)	39.6 (11.9–336.9) (n = 23)	514.1 (40.3–1194.5) (n = 35)	Kruskal Wallis— <0.0001 HC vs MSM— <0.0001 HC vs SM— <0.0001 HC vs CM— <0.0001 MSM vs SM— NS MSM vs CM— NS SM vs CM— 0.0235
Angiopoietin-2 (pg/mL)	752 (47–1340) (n = 59)	1952 (1055–2788) (n = 64)	2106 (1032–3213) (n = 23)	3,651 (2,487–6,121) (n = 36)	Kruskal Wallis— <0.0001HC vs MSM— <0.0001 HC vs SM— <0.0001 HC vs CM— <0.0001 MSM vs SM— NS MSM vs CM— 0.0001 SM vs CM— 0.0054
Arginine (µmol/L)	85.8 (72.0–111.4) (n = 59)	45.8 (34.7–60.0) (n = 64)	49.8 (37.8–62.8) (n = 23)	41.1 (30.7–52.1) (n = 36)	Kruskal Wallis— <0.0001 HC vs MSM— <0.0001 HC vs SM— <0.0001 HC vs CM— <0.0001 MSM vs SM—NS MSM vs CM— NS SM vs CM— NS
Plasma arginase (pmol/mL/hr)	0.096 (0.062–0.191) (n = 78)	0.118 (0.081–0.269) (n = 68)	0.121 (0.093–0.244) (n = 60)	0.166 (0.112–0.268) (n = 43)	Kruskal Wallis—0.0079 HC vs MSM— NS HC vs SM— 0.0288 HC vs CM— 0.0016 MSM vs SM— NS MSM vs CM—0.0491 SM vs CM— NS
Plasma cell-free Hb (μM)	0.82 (0.41–1.57) (n = 60)	0.90 (0.49–1.38) (n = 64)	1.04 (0.58–1.31) (n = 23)	1.22 (0.79–1.81) (n = 36)	Kruskal Wallis —NS HC vs MSM— NS HC vs SM— NS HC vs CM— NS MSM vs SM— NS MSM vs CM— 0.0455 SM vs CM— NS

Data are presented as median (inter-quartile range) unless stated otherwise. Significance testing across groups was done by Kruskal Wallis, and pairwise comparisons were done by Mann-Whitney U tests unless stated otherwise. NS = non-significant (p > 0.05); NA = not applicable.

^a^Mean (standard deviation). Significance testing across groups by ANOVA and pairwise comparisons by Student T-test.

^b^Chi-square test of proportions.

**Table 2:** Correlations of malaria disease severity measures with arginase activity, soluble CD163 measurements, and IL-10 measurements.

		Plasma Arginase Activity				sCD163				IL-10			
Analyte		HC	MSM	SM	All Malaria	HC	MSM	SM	All Malaria	HC	MSM	SM	All Malaria
HRP-2	r	−0.27	0.05	0.19	0.11	0.13	0.31	0.27	0.28	*	0.61	0.63	0.49
	p	0.15	0.75	0.31	0.37	0.51	0.12	0.39	**0.09**		**0.002**	**0.01**	**0.002**
	n	31	41	31	72	28	26	12	38		24	15	39
Ang-2	r	0.13	0.21	0.47	0.36	−0.06	0.04	0.14	0.09	−0.09	0.26	0.30	0.32
	p	0.45	**0.09**	**<0.001**	**<0.001**	0.76	0.84	0.58	0.55	0.73	**0.08**	**0.07**	**0.003**
	n	37	62	54	116	31	32	18	50	15	45	37	82
Lactate	r	0.36	0.24	0.31	0.30	−0.001	0.51	0.48	0.52	0.23	0.53	0.26	0.41
	p	**0.005**	0.09	**0.004**	**<0.001**	0.99	**0.009**	**0.003**	**<0.001**	0.29	**<0.001**	0.15	**<0.001**
	n	57	49	84	133	37	25	36	61	23	39	33	72
Arginine	r	−0.34	−0.34	−0.32	−0.33	−0.14	−0.22	−0.07	−0.14	0.12	−0.54	−0.022	−0.42
	p	**0.013**	**0.006**	**0.016**	**<0.001**	0.40	0.22	0.78	0.34	0.55	**<0.001**	0.18	**<0.001**
	n	53	63	55	118	37	33	18	51	29	46	38	84
Arg/ADMA	r	−0.47	−0.23	−0.49	−0.36	−0.34	−0.08	−0.44	−0.22	0.29	−0.35	−0.11	−0.26
	p	**<0.001**	**0.070**	**<0.001**	**<0.001**	**0.040**	0.67	**0.060**	0.13	0.11	**0.020**	0.49	**0.020**
	n	53	63	55	118	37	33	18	51	29	46	38	84
Hb	r	0.02	−0.01	−0.03	−0.06	−0.01	−0.06	−0.07	0.11	−0.37	0.06	0.28	0.11
	p	0.87	0.91	0.76	0.42	0.95	0.71	0.61	0.28	**0.050**	0.70	**0.09**	0.31
	n	77	66	102	168	54	41	51	92	29	45	38	83
Plt	r	0.02	−0.08	0.01	−0.05	0.14	−0.16	−0.02	−0.09	−0.20	**−0.26**	−0.25	−0.28
	p	0.89	0.50	0.91	0.48	0.29	0.31	0.89	0.35	0.30	**0.09**	0.14	**0.01**
	n	78	65	100	165	55	40	51	91	29	**45**	36	81

^*^There were not enough HC subjects with data for analysis.

r = rho (correlation coefficient); p = p-value for correlation; n = number of observations for each correlation test.

Correlations with a p value < 0.10 are noted by bold print.
