# Supplementary material for: Monocyte polarization in children with falciparum malaria: relationship to nitric oxide insufficiency and disease severity
Source: Sci Rep. 2016 Jul 7;6:29151. doi: 10.1038/srep29151 (PMC4935839; doi:10.1038/srep29151)
Supplement: Supplementary Information [file srep29151-s1.pdf]

Supplementary Tables for Manuscript:

Full title: **Monocyte polarization in children with falciparum malaria: relationship to nitric oxide insufficiency and disease severity**

Condensed title: **Monocyte activation and malaria in children**

Authors: J. Brice Weinberg[1], Alicia D. Volkheimer[1], Matthew P. Rubach[1], Salvatore M. Florence[2], Jackson P. Mukemba[2], Ayam R. Kalingonji[2], Charles Langelier[3], Youwei Chen[1], Margaret Bush[1], Tsin W. Yeo[4], Donald L. Granger[5], Nicholas M. Anstey[4], Esther D. Mwaikambo[2]

Institutions: [1] Duke University and V.A. Medical Centers, Durham, North Carolina

[2] Hubert Kairuki Memorial University, Dar es Salaam, Tanzania

[3] University of California at San Francisco

[4] Menzies School of Health Research and Charles Darwin University, Darwin, Australia

[5] University of Utah and V.A. Medical Centers, Salt Lake City, Utah

Supplementary Table 1. Bivariate and multivariate logistic regression analysis of soluble CD163 as independently associated with increased odds of severe malaria

Variable	N	Bivariate OR (95% CI)	p value	Multivariate OR (95% CI)	p value
**Soluble CD163** [A]	94	2.54 (1.12-5.76)	0.025	4.68 (1.31-16.68)	0.017
**Lactate**	155	1.70 (0.89-3.27)	0.110	--	>0.05 [B]
**Hemoglobin**	203	0.84 (0.74-0.96)	0.012	--	>0.05 [B]
**Absolute Monocyte Count**	173	1.60 (0.89-2.85)	0.111	--	>0.05 [B]
**Weight**	204	0.97 (0.90-1.04)	0.396	--	>0.05 [B]
**Age**	205	0.98 (0.82-1.16)	0.780	--	>0.05 [B]
**Sex**	205	1.22 (0.69-2.17)	0.491	--	>0.05 [B]

Note that soluble CD163, lactate, and absolute monocyte count values were log-transformed for regression analysis. There were 52 observations included in multivariate model.

A = Based on bivariate analysis, IL-10 (OR 1.31 [95% CI 0.91-1.75]; p=0.067) and CCL22 (OR 0.46 [95% CI 0.22-0.95]; p=0.036) should have been included in the multivariate model. However, inclusion of either of these variables decreased the total number of observations in the final multivariate model to 28. No other cytokine/chemokines had p-values <0.15 on bivariate logistic regression.

[illegible]

Supplementary Table 2. Bivariate and multivariate logistic regression analysis of plasma IL-10 as independently associated with increased odds of severe malaria

Variable	N	Bivariate OR (95% CI)	p value	Multivariate OR (95% CI)	p value
**IL-10**	84	1.31 (0.98-1.75)	0.067	1.42 (1.03-1.96)	0.032
**Lactate**	155	1.70 (0.89-3.27)	0.110	--	>0.05 [B]
**Hemoglobin**	203	0.84 (0.74-0.96)	0.012	--	>0.05[B]
**Absolute Monocyte Count**	173	1.60 (0.89-2.85)	0.111	--	>0.05 [B]
**Weight**	204	0.97 (0.90-1.04)	0.396	--	>0.05 [B]
**Age**	205	0.98 (0.82-1.16)	0.780	--	>0.05[B]
**Sex**	205	1.22 (0.69-2.17)	0.491	--	>0.05 [B]

Note that IL-10, lactate, and absolute monocyte count values were log-transformed for regression analysis. There were 70 observations included in multivariate model.

A= Based on bivariate analysis, sCD163 (OR 2.54 [95% CI 1.12 - 5.76]; p=0.025) and CCL22 (OR 0.46 [95% CI 0.22 - 0.95]; p=0.036) should have been included in the multivariate model. However, inclusion of either of these variables decreased the total number of observations in the final multivariate model to 28. No other cytokine/chemokines had p-values <0.15 on bivariate logistic regression.

B = included in multivariate logistic regression analysis, but not retained in the final model due to p-value > 0.05 in multivariate analysis.

Supplementary Table 3. Bivariate and multivariate logistic regression analysis of arginase activity as independently associated with increased odds of severe malaria

Variable	N	Bivariate OR (95% CI)	p-value	Multivariate OR (95% CI)	p-value
**Arginase**	171	1.31 (0.91-1.89)	0.067	--	>0.05 [A]
**Lactate**	155	1.70 (0.89-3.27)	0.110	--	>0.05 [A]
**Hemoglobin**	203	0.84 (0.74-0.96)	0.012	0.83 (0.69-0.99)	0.045
**Absolute Monocyte Count**	173	1.60 (0.89-2.85)	0.111	--	>0.05 [A]
**Weight**	204	0.97 (0.90-1.04)	0.396	--	>0.05 [A]
**Age**	205	0.98 (0.82-1.16)	0.780	--	>0.05[A]
**Sex**	205	1.22 (0.69-2.17)	0.491	--	>0.05 [A]

Note that arginase, lactate, and absolute monocyte count values were log-transformed for regression analysis. There were 116 observations included in multivariate model.

A = included in multivariate logistic regression analysis, but not retained in the final model due to p-value > 0.05 in multivariate analysis.

Supplementary Table 4. Primers and probes

Gene	Forward Primer	Reverse Primer	Probe	Probe cat#	ProbeSeq
**NOS2a**	tgaggaccacatctaccagga	caggatgtcctgaacatagacct	67	4688660001	tgctggag
**ARG1**	acatcacagaagaaatctacaaaacag	tgctgtgttcactgttcgagt	60	4688589001	tggggaag
**ARG2**	cgggggactaacctatcgag	ttcaacaagatccagtgctga	73	4688961001	gctgagga
**GAPDH**	agccacatcgctcagacac	gcccaatacgaccaaatcc	60	4688589001	tggggaag
**HPRT1**	tgaccttgatttattttgcatacc	cgagcaagacgttcagtcct	73	4688961001	gctgagga

Primers and probes were designed using Universal Probe Library Assay Design Center on Roche website http://universalprobelibrary.com/

Probes were purchased from Roche Universal Probe Library, and primers.
